# Is hypnosis a valid alternative to spontaneous breathing general anesthesia for claustrophobic patients undergoing MR exams? A preliminary retrospective study

**DOI:** 10.1186/s13244-021-01020-7

**Published:** 2021-06-25

**Authors:** Stefania Rizzo, Nicole Ferrera, Emanuele Pravatà, Roman Guggenberger, Steven Stern, Filippo Del Grande

**Affiliations:** 1 Facoltà Di Scienze Biomediche, Università Della Svizzera italiana (USI), Via Buffi 13, 6900 Lugano, Switzerland; 2 Clinica Di Radiologia EOC, Istituto Di Imaging Della Svizzera Italiana (IIMSI), Ente Ospedaliero Cantonale, Via Tesserete 46, 6900 Lugano, Switzerland; 3 Medico Indipendente, FMH Medicina Interna Generale, AFC Ipnosi Medica SMSH (Societé Médicale Suisse D’Hypnose), Hergiswil, Switzerland; 4 Istituto Di Neuroscienze Cliniche Della Svizzera Italiana, Ente Ospedaliero Cantonale, Via Tesserete 46, 6900 Lugano, Switzerland; 5 Institut Für Diagnostische Und Interventionnelle Radiologie, Universitäts Spital Zürich, Zurich, Switzerland; 6 Centre of Data Analytics, Bond University, Robina, Australia

**Keywords:** MRI, Hypnosis, Claustrophobia, Sedation

## Abstract

**Background:**

The purpose of our retrospective study was to assess the termination rate and the image quality of MR exams performed in claustrophobic patients under medical hypnosis, as compared to patients undergoing MR under spontaneous breathing general anesthesia.

**Methods:**

Our study was approved by the ethics committee. The “hypnosis group” included consecutive patients that had previously interrupted an MR exam because of claustrophobia. The “control group” included patients undergoing MR under pharmacologic sedation. Two experienced radiologists assessed, randomly, independently and blinded the image quality of the two groups using a symmetrical Likert scale: 0 = non-diagnostic images; 1 = bad image quality; 2 = fair image quality; 3 = good image quality; 4 = very good image quality. Descriptive statistics was performed.

**Results:**

Eighty patients were included, equally distributed between the two groups. Every patient was able to complete the MR exam. Ratings 3 and 4 represented the majority of ratings. Both readers rated the MR exams with score 3 or 4 in 66.25% (53/80) of MR exams. Only 5% (4/80) of MR exams were rated below score 2. The majority of the MR exams showed good or very good image quality. No significant difference was found in image quality between the two (*p* = 0.06) groups. The agreement between the two readers according to the *k* score was 0.105.

**Conclusions:**

Medical hypnosis is a valid alternative to spontaneous breathing general anesthesia in patients unable to undergo MR due to claustrophobia, allowing good quality images.

## Key points

- Medical hypnosis may help patients suffering from claustrophobia to undergo MR examination
- MR under medical hypnosis may be accomplished without impairing image quality
- Medical hypnosis can be considered a valid alternative to pharmacological sedation when performing MR in patients suffering from claustrophobia

## Background

Claustrophobia is a relatively frequent condition in the general population and, in the most severe forms, people who suffer from claustrophobia cannot accept the perception of not moving or not changing position freely and this can even culminate in a panic attack [1–6]. The incidence of claustrophobia ranges from 1 to 15% in patients scheduled for magnetic resonance (MR) examinations and some patients are not able to complete the MR exam without external support such as pharmacologic sedation [2, 6, 7].

In clinical practice, MR exams of such patients are usually performed under spontaneous breathing general anesthesia or conscious sedation with oral or low-dose intranasal benzodiazepines [8–11]. However, non-pharmacological techniques could sometimes be a preferred option.

Among the non-pharmacologic approaches in managing acute stressful situations, medical hypnosis is currently used in the Emergency Room and Internal Medicine departments [12–15]. To the best of our knowledge, there are only very few reports in the literature on the use of medical hypnosis to help patients suffering from claustrophobia to undergo an MR exam [16–19]. In our Radiology Department, hypnosis techniques are routinely proposed as an alternative to pharmacologic sedation to help patients experiencing claustrophobia to complete a scheduled MR.

The purpose of our study was to retrospectively assess the termination rate and the image quality of MR exams performed under medical hypnosis in patients experiencing claustrophobia, as compared to patients undergoing MR under spontaneous breathing general anesthesia**.**

## Methods

### Study design

Our single-center retrospective study was approved by the local ethics committee, with waiver of informed consent. One medical doctor (FDG) selected 40 patients who underwent an MR exam under hypnosis between November 2015 and February 2019 from our patient database (“*hypnosis group*”). Only patients older than 18 years and with a previously interrupted MR exam in the same body part because of claustrophobia were included. Forty consecutive patients older than 18 years who underwent MRI under spontaneous breathing general anesthesia because of claustrophobia between October 2018 and February 2019 were included as a control group.

MR exams were performed on a clinical wide-bore 3 T MR (MAGNETOM Skyra, Siemens Healthcare, Germany) in supine position. MR protocols, field of views, and RF coils were adapted to the type of MR exam and to the clinical indication.

### Medical hypnosis

Hypnosis was performed by a medical doctor board-certified in internal medicine and in medical hypnotherapy (N.F.). Following the exam scheduling, the hypnotherapist directly contacted every patient to explain the procedure and to ask if the patient was willing to undergo the MR exam under hypnosis. On the day of the MR exam, patients were accepted in the MR section 30 min. before the actual scheduled time, in order to induce the hypnotic trance. The hypnotherapist induced the hypnotic trance outside the MR room, by inviting the patient to follow guided meditation. The patient was allowed to choose the topic and was free, at any time, whether or not to follow the guided meditation. The hypnotherapist was outside the MR room and constantly in contact by microphone with the patient to maintain the hypnosis throughout the MR exam. None of the patients received anxiolytic medications.

### Spontaneous breathing general anesthesia

Patients in the “*control group*” fasted 8 h before the MR exam. Patients were scheduled 45 min before the MR exam time without any premedication. Pharmacological sedation was performed by board-certified anesthesiologists from the department of anesthesiology of our hospital. The standard protocol for pharmacologic sedation was as follows: Propofol 2 mg/kg i.v., Fentanyl 2 mcg/kg i.v. for induction. Propofol 6–8 mg/kg/h i.v. for maintenance of sedation. If intubation was indicated, Rocuronium 0.5 mg/kg was added.

### MR exam termination

The MR exam was considered complete if the standard MR protocol defined before the MR exam was finished and, if needed, additional sequences were performed.

### Image analysis

Two experienced radiologists (S.R., E.P.) with, respectively, 15 years and 12 years of experience in reading MR exams, assessed, randomly, independently and blinded, the quality of the images of the two groups using a symmetrical Likert scale (0 = non-diagnostic images; 1 = bad image quality; 2 = fair image quality; 3 = good image quality; 4 = very good image quality). The images were evaluated on a picture archiving and communication system workstation (PACS) (Philips Intellispace PACS, vers. 4.4.543.7, Philips Healthcare Informatics, Inc, Forster City, USA).

### Statistical analysis

Statistical evaluations and computations were performed with the support of R version 3.5 with the polr packages. The overall consistency between the reader ratings and the equality of the rating distributions for each reader was assessed. Overall consistency was compared using exact agreement as well as agreement to within one category. Differences in the scan-rating distributions between patients and controls were assessed using a proportional odds logistic regression model for comparing ordinal outcomes. Since the pelvis MR exams were rated lower in image quality and were unevenly distributed, an additional analysis was performed without pelvis MR exams on both groups. For completeness of the analysis, the comparison between ratings for the two groups was repeated after exclusion of ratings that showed a difference higher than 2 categories between the 2 readers, and also after pairing together the ratings 0 and 1, and the ratings 3 and 4. *k* values were calculated according to Landis and Koch and were: *k* between 0 and 0.2 represents slight agreement; between 0.21 and 0.4 represents fair agreement; between 0.41 and 0.60 represents moderate agreement; between 0.61 and 0.80 represents good agreement; and between 0.81 and 1.00 represents excellent agreement [20].

*p* values < 0.05 were considered significant.

## Results

Between December 2015 and February 2019, we included 40 patients in the hypnosis group (15 men, 25 women, mean age 60, range 30–86) and 40 patients in the control group (14 men, 26 women, mean age 61 range 33–88). The two groups were matched for gender (*p* = 0.907), age (*p* = 0.202), and type of MR exam (*p* = 0.106).

The MR exams in the hypnosis group were: 14 brain MRs; 2 cervical spine MRs; 8 lumbar spine MRs; 1 Hip MR; 6 whole spine MRs; 5 pelvis MRs; 3 shoulder MRs, 1 brachial plexus MR.

The MR examinations in the control group were: 15 brain MRs; 4 brain and spine MRs; 3 cervical spine MRs; 1 thoracic spine MR; 9 lumbar spine MRs; 1 Hip MR; 1 neck MR; 3 whole spine MRs, 2 aorta angiography MRs; 1 pelvis MR.

All patients of both groups were able to complete the MR examination. No adverse events were recorded.

Table 1 summarizes the cross-tabulation of all 80 scans, indicating the frequencies of all the possible pairs of reader ratings. Ratings 3 and 4 represent the great majority of ratings. Both readers rated the MR exams with score 3 or 4 in 66.25% (53/80) of MR exams. Only 5% (4/80) of MR exams were rated below score 2. Identical ratings of the two readers and ratings within one rating category of each other were present in 41.25% (33/80) of MR exams and in 92.5% (74/80) of MR exams, respectively. Of the 6 MR exams where the readers disagreed by more than 1 category, 4 were patients in the hypnosis group and 2 were controls. The agreement between the two readers according to the *k* score was 0.105.

**Table 1:** Cross-tabulation of the 80 scans, indicating the frequencies of the pairs of reader ratings for MR image quality

Reader 1 rating		Reader 2 rating			
	0	1	2	3	4
0	–	–	–	–	–
1	–	–	1	–	–
2	1	–	6	7	1
3	–	1	7	14	16
4	–	1	2	10	13

**Fig. 1:**
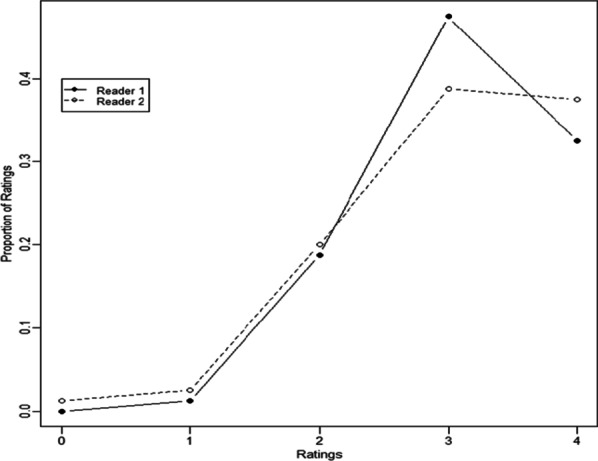
Distribution of ratings for reader 1 and reader 2

Figure 1 shows the distribution of ratings for each reader, confirming that neither reader systematically gave higher or lower ratings overall (however, reader 1 was slightly less likely to give a 4 and instead preferred a 3).

**Fig. 2:**
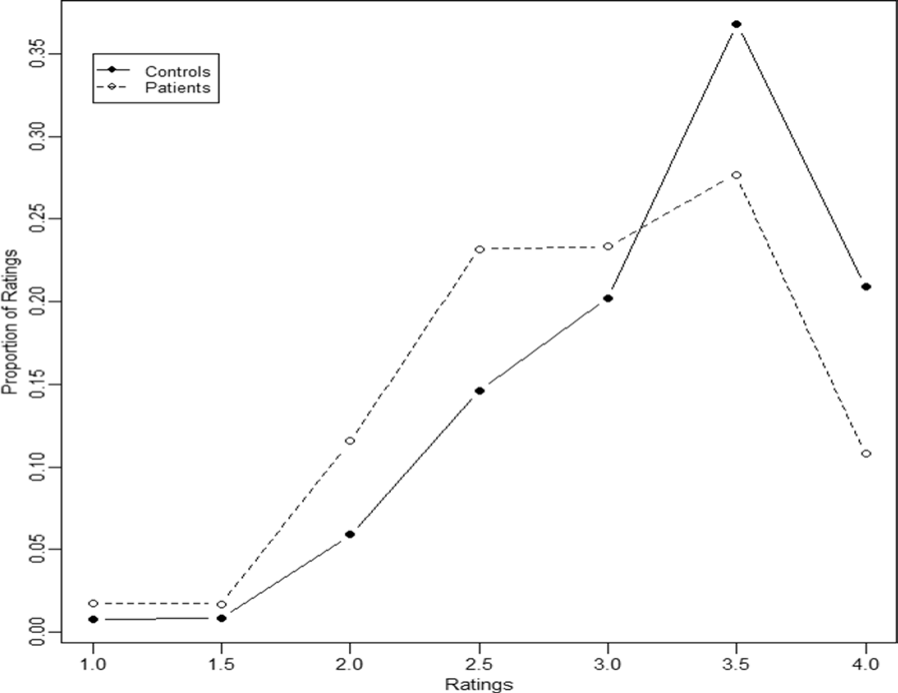
Distributions of average reader ratings

**Fig. 3:**
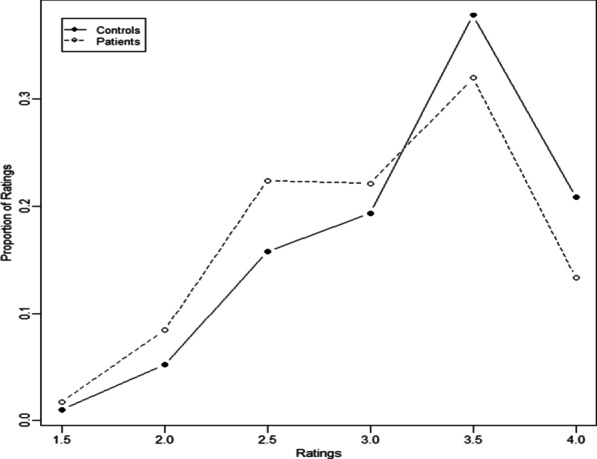
Distributions of average reader ratings (after exclusion of pelvis MR examinations)

Figure 2 shows the distributions of average reader ratings for the two groups showing a slight shift toward lower ratings for the patient group. The slight shift was not statistically significant (*p* value 0.06). By excluding MR exams of the pelvis, the *p* value for any difference in the ratings between hypnosis and control group was *p* = 0.21 (Fig. 3).

Finally, given the lack of clinical significance between ratings of 3 and 4 and ratings of 0 and 1, the above analysis was repeated with these pairs of categories combined. For this categorization, the readers’ ratings agreed exactly in 73.75% (59/80) of MR exams, and in only 2.5% (2/80) of MR exams did their ratings disagree by more than 1 category. The *p* value was 0.12 between the hypnosis and control groups and *p* = 0.38 when the pelvis MRs were excluded. The agreement between the two readers according to the *k* score was 0.254.

## Discussion

Our results indicate that medical hypnosis may allow patients experiencing claustrophobia to complete an MR exam without the need for general anesthesia. When compared to MR exams performed under spontaneous breathing general anesthesia, we did not record any statistically significant image quality degradation in the hypnosis group, although the distributions of average reader ratings for the two groups showed a slight shift toward lower ratings in the hypnosis group. As such, medical hypnosis can be an interesting non-pharmacologic option to overcome MR-related claustrophobia, at least in selected patients that are willing to take an active part in controlling claustrophobia and/or are not willing to undergo pharmacological sedation.

Interestingly, we found that the MR examinations of the pelvis were given the lowest average rating among all categories and the majority of the pelvis MRs were in the hypnosis group. (Five were assigned to the hypnosis group and 1 was in the control group.) This may account for a confounding effect of having more MRs of the pelvis in the hypnosis group than in the control group. Indeed, after excluding pelvic MRs, the shift toward lower readings for the patient group improved considerably. We hypothesize that the shift toward lower ratings in pelvis MRs of the hypnosis group may be due to bowel motion artifacts in the hypnosis group. (No medication was administered to suppress bowel motion in 4 out of 5 patients in the hypnosis group.) Furthermore, the i.v. administration of propofol and fentanyl during pharmacologic sedation could decrease bowel motion, thus leading to MR image quality improvement [21].

When scores 3 and 4 and 0 and 1 were grouped together, which we considered clinically irrelevant, our readers agreed exactly in almost ¾ of the cases, but at the same time the kappa value was considered fair (*k* = 0.254). This is probably because the *k* value depends not only on the degree of agreement, but also on the degree of evenness of spread across the categories. In our case, the vast majority of the ratings were 3 s or 4 s, so very uneven across the values 0 to 4.

Claustrophobia is a recurrent problem in MR departments and can lead to work-flow disruption and inefficient use of MR machines. According to a cohort study of more than 55,000 patients, between 1 and 15% of the patients undergoing MR suffer from claustrophobia and require sedation to complete the MR exams [2]. The usual way to overcome claustrophobia is to undergo the MR exam either under spontaneous breathing general anesthesia, or under conscious sedation with oral or intranasal benzodiazepine administration. Both techniques have some drawbacks. Spontaneous breathing general anesthesia requires a longer hospital stay to control the awakening and is more expensive due to the anesthesiology support. According to previous data, conscious sedation with intranasal benzodiazepine administration is an effective and safe option for patients suffering from claustrophobia without image quality impairment and with only a minor impact on the work-flow [8, 10, 11]. According to the prescribing information of the Food and Drug Administration, the most common adverse reactions of intranasal benzodiazepine administration are somnolence, headache, nasal discomfort, throat irritation, rhinorrhea, and drug interaction. Furthermore, after intranasal benzodiazepine applications, patients are not allowed to drive back home and/or perform dangerous activities such as operating machinery [22]. As a non-pharmacological technique, hypnosis has no side effects, patients are allowed to drive back home alone and are fully active as soon as the MR exam is over.

To the best of our knowledge, the application of hypnosis for patients suffering from claustrophobia and undergoing MRI is described only in one case report and two small case series. Friday and colleagues reported ten patients suffering from claustrophobia that were able to successfully complete an MR exam with the support of medical hypnosis. The authors described the hypnotherapist entering the MR examination room and applying pressure in different body parts of the patient. Unlike our approach, this hypnotic technique substantially increases the time needed for one MR exam [16]. We therefore induced the hypnotic trance outside the MR exam room, without interfering with the normal MR-schedule. Only when hypnosis had been induced was the patient transported to the MR room to start the MR exam. The hypnotic trance was maintained by microphone with the hypnotherapist outside the MR room, using the same technique described by Simon [18]. We are aware of only one other study that reported 15 out of 16 patients suffering from claustrophobia that were able to complete the MR exam under hypnosis [17]. None of the studies provides any information on image quality.

Our study has some limitations. First, the number of patients was relatively small. However, this was a preliminary retrospective study aiming to assess the non-inferiority of image quality of MR examinations between medical hypnosis and spontaneous breathing general anesthesia. Second, the included patients did not fill out any validated claustrophobia questionnaire. However, every included patient had previously interrupted an MR due to claustrophobia, indicating that there was an objective difficulty in handling this stressful situation. Third, the patients were included on a voluntary basis; while this could potentially lead to selection bias, hypnosis is effective only with actively cooperative patients and a random selection would be difficult to implement for such a procedure. Fourth, the study was not designed to focus on images from a specific regional anatomy, although the two groups matched regarding MR exam type (no statistical differences) and as such were comparable.

In conclusion, MR exams performed under medical hypnosis do not significantly compromise image quality compared to MR exams performed under spontaneous breathing general anesthesia. As such, in experienced hands, medical hypnosis is a promising and valid alternative to spontaneous breathing general anesthesia in patients unable to undergo MR due to claustrophobia, if they prefer non-pharmacologic sedation and if they are able and willing to cooperate with the hypnotherapist.

## Data Availability

The datasets used and analyzed during the current study are available from the corresponding author on reasonable request.

## Abbreviations

MR: Magnetic resonance
PACS: Picture archiving and communication system
